# Anterior column realignment via a minimally invasive hybrid approach in adult spinal deformity surgery: a short-term retrospective study

**DOI:** 10.1186/s12891-023-07106-1

**Published:** 2023-12-19

**Authors:** Da Peng Feng, Ming Quan Liu, Wei Zhang, Jia Qi Wang, Zheng Wei Li

**Affiliations:** 1 The Second Affiliated Hospital of Dalian Medical University, Liaoning, People’s Republic of China; 2 Sanbo Brain Hospital, Capital Medical University, Beijing, People’s Republic of China

**Keywords:** Musculoskeletal diseases, Lumbar lordosis, Surgical procedures, Spinal fusion, Anterior longitudinal ligament

## Abstract

**Background:**

Anterior column realignment (ACR) is a novel surgical method for correcting spinal sagittal balance. meanwhile, oblique lumbar interbody fusion (OLIF) and anterior lumbar interbody fusion (ALIF) are considered minimally invasive surgical methods through natural anatomical space. This study aimed to explore the corrective effects and clinical outcomes of OLIF or ALIF combined with ACR technology in patients with adult spinal deformity (ASD).

**Methods:**

We retrospectively analyzed patients with sagittal imbalance who received OLIF and/or ALIF and ACR treatment from 2018 to 2021. Surgical time and intraoperative bleeding volume are recorded, the corrective effect is determined by the intervertebral space angle (IVA), lumbar lordosis (LL), the sagittal vertical axis (SVA), clinical outcome is evaluated by preoperative and final follow-up visual analog pain score (VAS), Japanese orthopedic association scores (JOA) and complications.

**Results:**

Sixty-four patients were enrolled in the study, average age of 65.1(range, 47–82) years. All patients completed 173 fusion segments, for 150 segments of ACR surgery. The operation time of ALIF-ACR was 50.4 ± 22.1 min; The intraoperative bleeding volume was 50.2 ± 23.6 ml. The operation time and intraoperative bleeding volume of single-segment OLIF-ACR was 66.2 ± 19.4 min and 70.2 ± 31.6 ml. At the follow-up of 6 months after surgery, the intervertebral space angle correction for OLIF-ACR and ALIF-ACR is 9.2° and 12.2°, the preoperative and postoperative lumbar lordosis were 16.7° ± 6.4°and 47.1° ± 3.6° (p < 0.001), VAS and JOA scores were improved from 6.8 to 1.8 and 7.8 to 22.1 respectively, statistically significant differences were observed in these parameters. The incidence of surgical related complications is 29.69%, but without serious complications.

**Conclusion:**

ACR via a minimally invasive hybrid approach for ASD has significant advantages in restoring local intervertebral space angulation and correcting the overall sagittal balance. Simultaneously, it can achieve good clinical outcomes and fewer surgical complications.

**Supplementary Information:**

The online version contains supplementary material available at 10.1186/s12891-023-07106-1.

## Background

Adult spinal deformity (ASD) is an increasingly serious health problem in the expanding portion of the global population aged older than 65 years. Meanwhile, Non-operative management of adult spinal deformity has been demonstrably ineffective, perhaps only benefiting for milder spinal deformities [1]. Hence, surgical treatment is the most appropriate way for those who want to improve their quality of life. The conventional surgical procedures for adult spinal deformities are mostly osteotomies and orthopedic procedures, including Smith-Petersen osteotomy (SPO), pedicle subtraction osteotomy (PSO), and vertebral column resection (VCR). Although osteotomies are widely performed in clinical practice and their effectiveness has been demonstrated by clinical studies [2]. However, osteotomy has many disadvantages, such as long operation time, high risk of nerve damage, and high blood loss [3–6]. The percentage of postoperative complications increases significantly with the extent of the osteotomy, with studies showing a 28% complication rate for SPO and a 61% complication rate for VCR [7].

Recently, with the development of minimally invasive surgical techniques, numerous new surgical techniques have been employed in the treatment of spinal deformities in adults. Oblique lateral interbody fusion (OLIF) and anterior interbody fusion (ALIF) are currently the most popular procedures in spinal surgery [8]. The two procedures can restore the physiological lumbar lordosis, return the height of the intervertebral space, and have low surgical complications [9, 10]. But, for severe spinal deformities, due to obstruction of the anterior longitudinal ligament (ALL) and hypertrophic osteophytes, it is extremely difficult to restore sagittal balance through simple interbody fusion cage implantation, and even increases the possibility of endplate injury.

Anterior column realignment (ACR), as an alternative to osteotomy, corrects sagittal imbalance by releasing the ALL and inserting a wedge-shaped intervertebral fusion cage to increase the angle of the intervertebral space [11, 12]. To the best of our knowledge, there are no relevant literature to reveal the corrective effects of OLIF and/or ALIF combined with ACR technology on patients with ASD. Therefore, our team combines ACR technology with ALIF or OLIF, believing that this technology can effectively restore sagittal balance while reducing surgical complications. In this study, we aimed to explore the corrective effects and clinical outcomes of OLIF or ALIF combined with ACR technology in patients with ASD.

## Methods

### Sample size

At the Second Affiliated Hospital of Dalian Medical University, since 2018, OLIF or ALIF is a routine surgical procedure for lumbar degenerative diseases, excluding ① Lumbar spinal stenosis caused by posterior bony structure hyperplasia, ② Lumbar spondylolisthesis of degree II or greater, ③ Dissociated disc herniation, ④ Local kyphosis deformity caused by fractures, tumors, etc. Meanwhile, for patient with PI-LL mismatch > 10°, the ACR technique is performed simultaneously with surgery.

In this study, we retrospectively analyzed patients with sagittal imbalance who received OLIF and/or ALIF and ACR treatment from 2018 to 2021 at our hospital. All patients were included in this study based on the following inclusion and exclusion criteria.

#### Inclusion and exclusion criteria

The inclusion criteria include: ① Patients with clinically diagnosed adult spinal deformity. ② Radiographic assessment: SVA > 40 mm, PI-LL mismatch > 10°. ③ Symptoms are obvious and received OLIF and/or ALIF and ACR treatment. ④ follow-up time than 6 months.

The exclusion criteria include: ① Lack of information including demographics, surgical data, radiographic data. ② Adolescent idiopathic scoliosis patients who developed later in adulthood. ③ Trauma, inflammation, tumor, or neuromuscular origin; ④ Severely spinal sagittal imbalance (SVA > 20 cm or PI-LL > 40°); ⑤ with previous lumbar surgery.

### Surgical technique

The anterior longitudinal ligament (ALL) can enhance the stability of the spine and as a barrier to prevent anterior dislodgement of the interbody cage. However, concerning sagittal deformity correction, ALL is also the main obstacle to against anterior column lengthening and deformity correction. Therefore, ACR technology includes the release of ALL and lateral ligament complex, and the placement and fixation of a hyperlordotic cage to restore LL.

All the operations were performed by the same team in our hospital. All patients were given general anesthesia and were performed by Minimally invasive hybrid approach. All patients, excluding one or two segment surgeries, underwent ACR and posterior percutaneous screw fixation one stage.

**Fig. 1:**
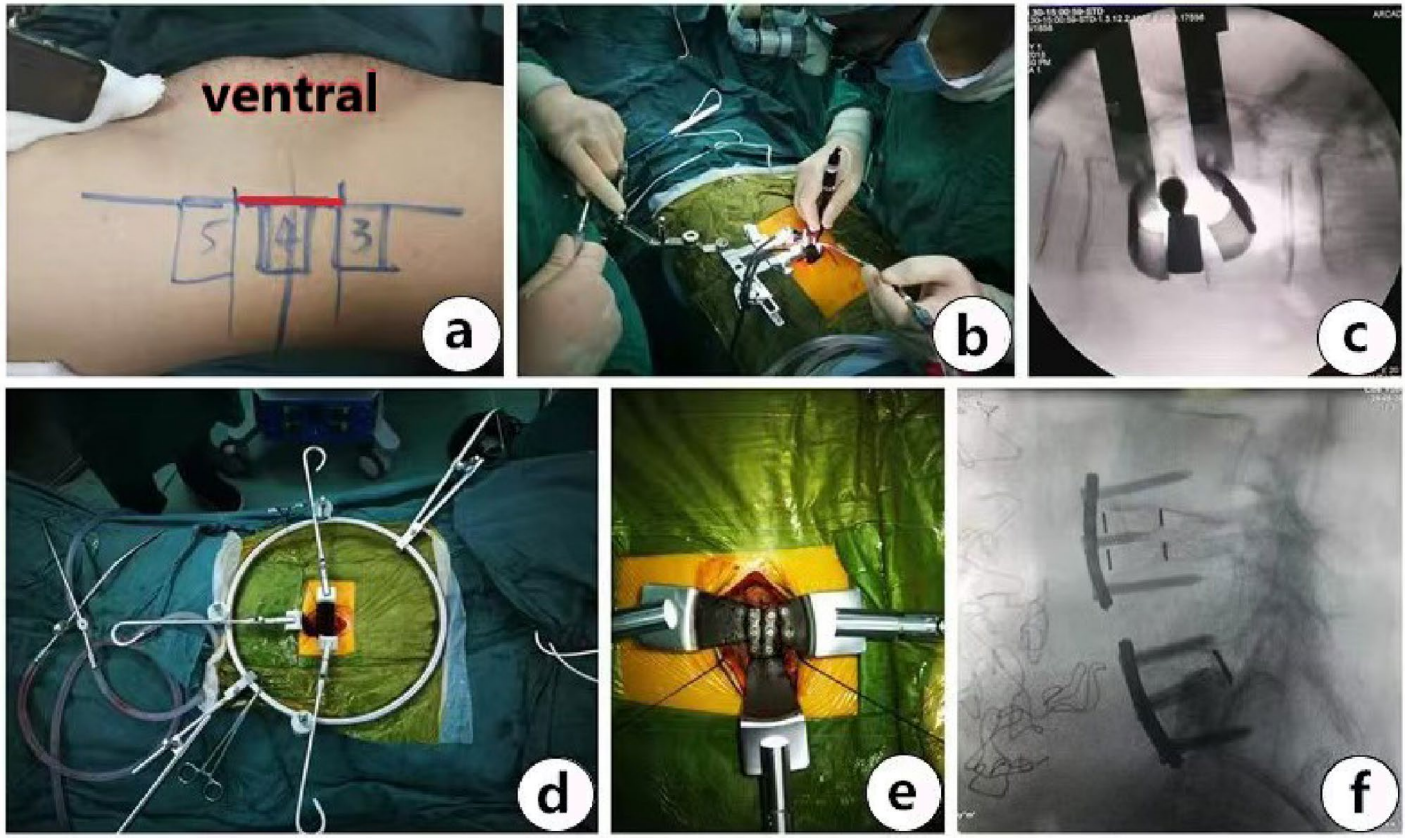
(**a**) Surgical incision of OLIF (L3-5); (**b**) Working chanel of OLIF; (**c**) Test model to open the intervertebral space; (**d**) The retracter of ALIF (**e**, **f**) the cage fixed to adjacent vertebral body

In the pre-operative stage of the procedure, the patient was placed in the standard right lateral position (RLP). Take a 3–5 cm surgical incision at the anterior projection of the vertebral body (Fig. 1a). Use the retroperitoneal approach to access the intervertebral disc space, and then the retractor is placed to expose the intervertebral disc in front of the posterior retractor blade (Fig. 1b). Gentle dissection is performed to identify and separate the plane between ALL and the anterior structure. Two-thirds of all anterior intervertebral discs must be removed to facilitate ALL release. Release the anterior longitudinal ligament and anterior vascular structure, a narrow abdominal retractor is inserted between them, and then release ALL with a long-handled scalpel. Use a reamer to break through and disconnect the contralateral annulus. After that, thoroughly expand the intervertebral space during the test model (Fig. 1c). Subsequently, the intervertebral fusion cage (Medtronic Sofamor Danek USA, lnc) (Size:16 mm*50 mm*6/12/18 DEG) loaded with allogeneic bone (Shanxi Aurui) will be implanted using standard techniques.

For the obstruction of iliac crest, L5/S1 segment cannot be performed at the same incision. Usually, the anterior approach is via the abdominal or retroperitoneal approach, require the patient to take a supine position and make a new surgical incision in the middle of the lower abdomen, also need specialized anterior traction instruments to help expose the intervertebral space (Fig. 1d). ALL can be removed under direct vision, Considering that ALL release will make the fusion cage move forward, it is very important to Internal fixation the implants to the vertebral body with 1 or 2 screws to reduce the risk of implant movement (Fig. 1e and f ). For patients with lumbar spondylolisthesis who require posterior reduction, fixing the cage to only 1 vertebral body is recommended.

The surgical decompression technique of ALIF is the same as anterior cervical discectomy and fusion (ACDF), posterior longitudinal ligament and disc herniation can be removed visually. For OLIF, intraspinal decompression can only be achieved through inclining working channel or inserting a camera system into the operating channel, such as percutaneous transforaminal endoscopic discectomy (PTED). Meanwhile, for patients with anterior fusion in the first stage, if the postoperative symptoms do not improve significantly, the posterior decompression can be performed in the second stage through the pedicle screw channel.

Posterior fixation usually uses percutaneous pedicle screw fixation (PPSF). For patients who undergoing multi-segmental surgery, due to anterior screw fixation, usually not requiring all surgical vertebral body to undergo posterior fixation, both ends and middle 1 or 2 vertebrae are sufficient. Meanwhile It is necessary to supplement bone cement reinforcement for patients with osteoporosis, especially when the T-value of bone density is less than − 2.5.

### Perioperative management

Pre-operation: All patients underwent X-ray, CT, and lumbar MRI examinations before surgery to determine the surgical strategy. Polyethylene glycol-electrolyte solution used in preoperative gut cleaning 24 h before surgery, and if necessary, soap solution should be used to clean the enema 12 h before surgery For ALIF surgery patients.

Post-operation: On the first or second day after the operation, lumbar spine x-rays was repeated with the duration postoperative activity determined by the patient’s status. Most patients were able to walk after surgery day 1–3, protected by a lumbar brace, and discharged on the 3rd day after surgery. Methylprednisolone (Pfizer Manufacturing Belgium NV) 120 mg intravenous drip once every day and continuous for 3 days. Non-steroidal anti-inflammatory drugs are usually given as short-term postoperative analgesics to patients when needed. prevention of lower limb venous thrombosis by using rivaroxaban, starting at 48 h after surgery, take 10 mg orally once a day until 2 weeks after surgery. For patients with osteoporosis, standardized treatment of osteoporotic osteoporosis is necessary after surgery.

### Data collection

The patient’s clinical and radiological information is obtained by accessing the electronic record system. The standard demographics, including age, sex. Several variables pertinent to the operative data were recorded for each patient, including operative time, estimated blood loss (EBL), method for interbody fusion. The imaging data mainly includes: pelvic incidence (PI), lumbar lordosis (LL), Intervertebral space angle of the surgical segment (IVA); sagittal vertical axis (SVA). Intraoperative or postoperative complications were recorded.

At 1-, 3-, and 6-months postoperative follow-up, frontal and lateral radiographs of the lumbar spine were obtained to measure IVA, LL, and SVA. CT examinations were performed at 3 months postoperatively to assess fusion and internal fixation stability. Pre-operative and post-operative follow-ups were performed using the VAS score and lumbar JOA score to assess the clinical outcome.

### Data analysis

The SPSS 25.0 software was used to analyze the data. The statistical results were described, with continuous variables shown as means and standard deviations, and classification shown as a percentage. Kolmogorov-Smirnov test was used to evaluate the normality of the variables, after that the paired t-test or Wilcox test was used to compare preoperative and last follow-up data. P < 0.05 was regarded as statistically significant.

## Results

A total of 64 patients (including 23 males and 41 females) were included the study, average age of 65.1(range, 47–82) years. All patients suffer varying degrees of intractable lumbago. Among them, 37 patients with 53 levels of lumbar disc herniation who conform to the symptoms of innervation zone. (L1-2 n = 2, L2-3 n = 5, L3-4 n = 9, L4-5 n = 24, L5-S1 n = 13); 21 patients with 25 levels of lumbar spinal stenosis (L2-3 n = 3, L3-4 n = 6, L4-5 n = 15, L5-S1 n = 1); 11 patients of 13 levels of degenerative spondylolisthesis (L3-4 n = 3, L4-5 n = 6, L5-S1 n = 3), according to the Meyerding classification of spondylolisthesis, 9 cases were grade I and 4 cases were grade II.

### Analysis of surgical details

We performed ACR via a minimally invasive combination approach for ASD, ALIF-ACR for L5-S1 segment and OLIF-ACR for L1-L5, effectively combining the advantages of both approaches with significant results. All 64 patients in this group completed ACR via a minimally, including 5 cases of OLIF-ACR alone and 3 cases of ALIF-ACR alone; In 56 cases of ALIF-ACR combined with OLIF-ACR, all patients completed 173 ALIF or OLIF fusion segments; A total of 150 segments of ACR were completed, including 91 segments of OLIF-ACR and 59 segments of ALIF-ACR.

Table 1 summarizes the bleeding and surgical time during different surgical approaches. The operation time of ALIF-ACR was 35–130 min, with an average of 50.4 ± 22.1 min; The intraoperative bleeding volume was 30–130 ml, with an average of 50.2 ± 23.6 ml. The operation time of single-segment OLIF-ACR was 42–100 min, with an average of 66.2 ± 19.4 min; The intraoperative bleeding volume was 30–150 ml, with an average of 70.2 ± 31.6 ml. The operation time of two-stage OLIF-ACR was 60–160 min, with an average of 96.2 ± 27.4 min; The intraoperative bleeding volume was 50–165 ml, with an average of 87.2 ± 38.6 ml; The operation time of three-segment OLIF-ACR was 68-142.5 min, with an average of 106.2 ± 37.3 min; The intraoperative bleeding volume was 79-152.5 ml, with an average of (117.2 ± 36.8) ml. percutaneous pedicle screw fixation with an average of (4.6 ± 1.66)nail; The operation time of PPSF was 40–250 min, with an average of (78.9 ± 52.4) min; The intraoperative bleeding volume was 40-470 ml, with an average of (130.6 ± 98.2) ml. All patients did not receive blood transfusions.

**Table 1:** Surgery-related data

Variable	Bleeding volume (ml)	Operation time (min)	n
ALIF-ACR	50.2 ± 23.6	50.4 ± 22.1	59
OLIF-ACR			
1	70.2 ± 31.6	66.2 ± 19.4	24
2	87.2 ± 38.6	96.2 ± 27.4	29
3	117.2 ± 36.8	106.2 ± 37.3	3
PPSF	130.6 ± 98.2	78.9 ± 52.4	64

PPSF: Percutaneous pedicle screw fixation

### Analysis of radiographic and clinical results

Table 2 summarizes the radiological evaluations of preoperative and postoperative radiographic date. There were significant differences in LL, IVA (ALIF-IVA; OLIF-IVA), SVA between before surgery and at the final observation. Among the 64 patients followed up, 55 underwent CT examination of the lumbar spine at 3 months postoperatively. In 53 of these cases, continuous trabecular formation between the upper and lower endplates was seen, achieving a fusion with a 96.2% fusion rate at 3 months postoperatively. The remaining 2 cases achieved bony fusion at 6 months postoperatively.

**Table 2:** Comparison of the preoperative and postoperative radiographic date

	Preoperative	Postoperative		Final follow-up	T/Z	P	*d*_*RM*_
LL (°)	16.7 ± 6.4		48.6 ± 10.7	47.1 ± 3.6	Z=-6.96	< 0.01	3.86
ALIF-IVA (°)	4.9 ± 2.5		22.7 ± 4.9	17.1 ± 3.6	T = 21.51	< 0.01	3.40
OLIF-IVA (°)	6.9 ± 3.1		18.8 ± 4.4	16.1 ± 3.3	T = 22.85	< 0.01	2.35
SVA (mm)	65.1 ± 10.1		29.5 ± 12.5	31.5 ± 14.3	Z = 6.95	< 0.01	-3.11

LL: lumbar lordosis; IVA: Intervertebral space angle of the surgical segment; SVA: sagittal vertical axis

As shown in Table 3 and 64 patients were followed up for 6–33 months with a mean of 17.7 ± 7.8 months. The mean preoperative VAS score was 6.8 ± 1.6, the mean postoperative score was 2.1 ± 0.7 and the mean score at the last follow-up s was 1.8 ± 0.8, achieving a 69.1% improvement rate before and after surgery, with a statistically significant difference (p < 0.01). The mean preoperative lumbar JOA score was 7.8 ± 2.9, the mean postoperative score was 19.1 ± 3.7 and the mean at the last follow-up s was 22 ± 3.9, achieving an improvement rate of 59.2% before and after surgery, a statistically significant difference (P < 0.01).

**Table 3:** Comparison of the preoperative and postoperative clinical outcomes

	Preoperative	Postoperative	Final follow-up	T/Z	P	*d*_*RM*_
VAS	6.8 ± 1.6	2.1 ± 0.7	1.8 ± 0.8	Z = 6.98	< 0.01	-2.28
JOA	7.8 ± 2.9	19.1 ± 3.7	22.1 ± 3.9	Z=-6.97	< 0.01	5.68

VAS: visual analog scale

## Complications

In our study, a total of 19 patients developed one or more complications, representing 29.69% of the patients. According to the Glassman classification criteria [13], major postoperative complications include nerve damage, vascular damage, and organ damage, and secondary complications included sensory impairment, motor impairment, hematoma, and incisional infection.

Four patients developed major complications after surgery, accounting for 6.25% of the patients. 2 patients presented with lower limb muscle weakness after surgery, one was a 58-year-old female who underwent L4-5 segment OLIF-ACR and developed right ankle muscle weakness after surgery, (preoperative muscle strength grade 4, postoperative muscle strength grade 2) muscle strength recovered to grade 4 after 3 months of rehabilitation; The other case was a 49-year-old male who underwent L5-S1 segmental ALIF-ACR and had lower extremity muscle weakness (preoperative grade 5, postoperative grade 1) which recovered to grade 3 after 9 months of rehabilitation; two patients had vascular injuries, one underwent L4-5 segmental OLIF-ACR with intraoperative damage to the segmental artery and one underwent L4-5 OLIF-ACR with an intraoperative partial tear of the common iliac artery which was repaired with silk sutures assisted by a vascular surgeon Repair.

There were 15 cases of secondary complications, accounting for 23.43% of the patients. 14 patients developed postoperative numbness in the inguinal region, anterior and lateral thighs, of which 11 recovered within 1 month after surgery and 3 gradually recovered between 3 and 6 months after surgery. 8 patients experienced left-sided hip flexion weakness, all of which recovered within 1 month after surgery. 11 patients developed temporary abdominal pain and bloating all of them recovered within 1 week after surgery.

All patients had no screw breakage and cage shifting within six months, only 7 cases of endplate collapse, but it also meets the standard for bone fusion.

## Case presentation

**Fig. 2:**
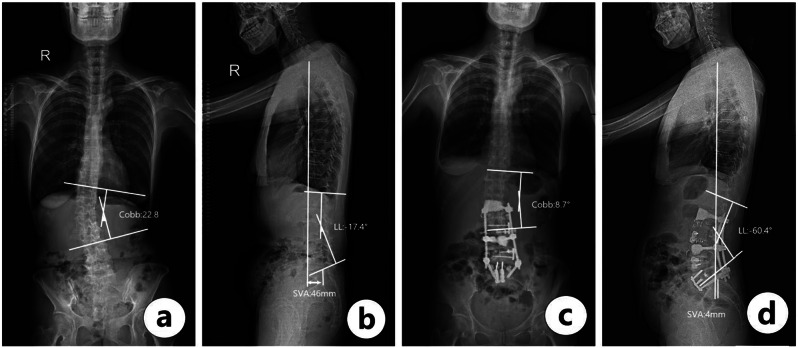
(**a**, **b**) Preparative frontal and lateral view radiographs (**c**, **d**) Postoperative frontal and lateral view radiographs

A 58-year-old woman with degenerative spinal deformities was indicated for anterior column reconstruction via a minimally invasive hybrid approach (Fig. 2). Figure 2a and b are full-standing X-rays before operation. Preoperative SVA = 46 mm, PI = 56.3°, LL = 17.4°, Cobb (T12-L3) = 22.8, | PI-LL |=42.9°. IVA (L2-3 = 5.8°; L3-4 = 8.3°; L4-5 = 12.0°; L5-S1 = 4. 2°)。Patients accepted L2-3 OLIF; L3-L5 OLIF-ACR; L5-S1 ALIF-ACR. Figure 2c, and Fig. 2d are full-standing X-rays after the operation. Postoperative SVA = 4 mm, LL = 60.4°, Cobb (T12-L3) = 8.7°. IVA (L2-3 = 7.2°; L3-4 = 17.8°; L4-5 = 15.3°; L5-S1 = 25.4°).

## Discussion

In this study, we combined OLIF and/or ALIF technology with ACR. 64 patients underwent this surgical procedure. By comparing imaging and clinical parameters, it was found that the minimally invasive mixed approach has significant advantages in restoring sagittal balance for adult spinal deformities, while effectively reducing surgical complications. And notably, due to the increase of contact area and the amount of bone grafting, the bone fusion rate reached 96.2% after 3 months.

ASD is a continuously progressive three-dimensional spinal deformity [14]. With the loss of lumbar lordosis as the disease progresses results in a forward shift of the patient’s trunk center of gravity and compensatory pelvic posterior rotation to maintain overall trunk balance, leaving the muscles of the low back in a state of chronic fatigue, resulting in intractable lower back pain and functional impairment [15]. Therefore, it is crucial to restore sagittal balance in the treatment of ASD [16]. But, the traditional treatment modalities such as Smith-Petersen osteotomy or PSO osteotomy are effective in restoring sagittal balance, but osteotomy is prone to a variety of complications such as excessive blood loss, neurological deficits and pseudoarthrosis [17]. Meanwhile, degenerative disease is often combined with spinal stenosis, The objective is to restore sagittal and coronal equilibrium while simultaneously decompressing the spinal canal and minimizing surgical complications [18].

In recent years, minimally invasive surgery has developed into an alternative method to avoid the complications of traditional open surgery, which can effectively reduce the incidence of complications related to the surgical approach and accelerate postoperative rehabilitation, and has become the development direction of surgery [19]. ACR is a new alternative method to treat ASD. ACR technology includes lumbar discectomy, anterior longitudinal ligament (ALL) disconnection, and wedge-shaped interbody fusion cage placement. If necessary, percutaneous, or open technology is used for posterior release and fixation to complete lumbar lordosis reconstruction [20, 21]. A systematic review by Cheung ZB [22], showed that at each ACR level, the increased range of focal segmental lordosis was 1–34°, and the average improvement range of LL was 12.7–39°. In our study, the average sagittal correction angle of each ACR segment was 10.3°, lumbar lordosis increased from 16.7° to 77.1°, and SVA improved from 65.1 mm to 29.5 mm. All imaging parameters were significantly improved, which was close to Cheung ZB.

ACR, as a minimally invasive alternative to osteotomy, achieved similar radiographic results as PSO and with significantly less estimated blood loss [23]. However, due to the release of anterior longitudinal ligament, there is a risk of injury to the autonomic nerve plexus, visceral organs, or large vessels. Although nerve injury may occur at any level, the femoral nerve injury rate is highest at the L4/5 level. It is important to distinguish between true motor weakness distributed along the femoral nerve and pain limiting weakness caused by hip flexion. The reported range of true motor weakness is 3.4–23.7%, the incidence of sensory abnormalities is 0.7–30%, and the reported range of numbness is 8.3–42.4% [24–26]. The commonly affected sensory nerves are the reproductive femoral nerve, the lateral femoral cutaneous nerve, and the anterior femoral cutaneous nerve. Most motor and sensory disorders are temporary and can be restored, with a recovery of 50% in 90 days and 90% in 1 year [27]. In this study, the incidence of complications was 29.69%, but most of them were secondary complications such as transient muscle weakness and abdominal distension (23.43%). Except for one case where muscle strength did not fully recover, the rest recovered within six months and no serious complications occurred.

ASD patients usually combined with lumbar spinal stenosis, intervertebral disc herniation and so on, nerve decompression is necessary during correction. Anterior approach interbody fusion is considered to be an effective treatment for degenerative disc disease (with or without neurological dysfunction) and lumbar spondylolisthesis [28]. In this study, we first performed effective decompression of the spinal canal using ALIF or OLIF techniques before performing ACR surgery. The symptoms of patients have been improved to varying degrees, VAS and JOA scores were improved from 6.8 to 1.8 and 7.8 to 22.1 respectively.

But OLIF and ALIF, as an indirect decompression technique, do not directly remove the posterior bony ligaments and hypertrophic ligaments, its decompression efficacy has been suspected. However, current meta-analysis shows that indirect and direct decompression similarly effective in patients with lumbar spinal stenosis and instability. Meanwhile, Indirect decompression also has advantages such as surgical time and tissue damage [29]. But, ACR is different from simple indirect decompression in that there is a possibility of accentuating foraminal or central stenosis during the expansion of the anterior column. Therefore, we advocate that all patients undergo anterior spinal canal decompression as much as possible, while avoiding excessive stretching of single segments, especially spinal canal stenosis caused by posterior ligament hyperplasia. At the same time, distraction technique is necessary during the posterior instrument fixation, although there may be a loss of corrective effect. Additional posterior decompression is still clinically effective in patients with reduced stenosis but still with calf pain and a positive straight leg raise test or femoral nerve stretch test after lateral approach lumbar fusion [30].

For patients with a degenerative disease, We believe that posterior decompression is necessary in cases of lumbar stenosis of grade D in the Schizas [31] classification, ligamentum flavum folding on MRI in overextension position, lumbar spondylolisthesis of degree II or greater and prolapsed lumbar disc herniation where effective disc removal is not possible anteriorly or laterally.

## Study limitations

First, the study lacks long-term follow-up results. Second, all patients completed ACR technology based on spinal canal decompression and fusion, which has an impact on ACR correction ability. Third, the surgical procedure is performed within the abdominal cavity and require surgeons to understand the anatomical structure, which will have a long learning curve for spinal surgeon. Fourth, the study has not analysis the homogeneity of included cases, such as BMI, TK, osteoporosis, etc. Additionally, Imaging parameters were measured simultaneously by two different observers, inevitably results in systemic errors in manual measurements.

## Conclusions

Our study confirms a minimally invasive hybrid access-based ACR procedure with satisfactory clinical outcomes. Although there are complications related to the surgical approach, such as such as vascular and anterior plexus injuries, but the surgical technique has significant advantages in restoring local intervertebral space angulation and correcting the overall sagittal balance. It also has the advantages of minimal muscle damage, low risk of nerve injury, low bleeding, and rapid post-operative recovery. We are confident that the ACR via a minimally invasive hybrid approach is a safe and effective strategy for ASD.

### Electronic supplementary material

Below is the link to the electronic supplementary material.

**Supplementary Material 1:** The database containing data with unanalyzed values

## Data Availability

Data cannot be provided due to identifying information of participants but. are available from the corresponding author on reasonable request.

## Abbreviations

ASD: Adult spinal deformity
ACR: Anterior column realignment
OLIF: Oblique lumbar interbody fusion
ALIF: Anterior lumbar interbody fusion
PI: Pelvic incidence
LL: Lumbar lordosis
IVA: Intervertebral space angle of the surgical segment
SVA: Sagittal vertical axis
VAS: Visual analog scale
SPO: Smith-Petersen osteotomy
PSO: Pedicle subtraction osteotomy
VCR: Vertebral column resection
ALL: Anterior longitudinal ligament
PPSF: Percutaneous pedicle screw fixation

## References

[1] Diebo BG, Shah NV, Boachie-Adjei O (2019). Adult spinal deformity. Lancet (London England).

[2] Heary RF (2004). Evaluation and treatment of adult spinal deformity. Invited submission from the Joint Section Meeting on disorders of the spine and peripheral nerves, March 2004 [J]. J Neurosurg Spine.

[3] Dorward IG, Lenke LG (2010). Osteotomies in the posterior-only treatment of complex adult spinal deformity: a comparative review[. J] Neurosurg Focus.

[4] Hyun SJ, Rhim SC (2010). Clinical outcomes and Complications after pedicle subtraction osteotomy for fixed sagittal imbalance patients: a long-term follow-up data [J]. J Korean Neurosurg Soc.

[5] Gill JB, Levin A, Burd T, Longley M (2008). Corrective osteotomies in spine Surgery. J Bone Joint surg American Volume.

[6] Bridwell KH, Lewis SJ, Edwards C et al. Complications and outcomes of pedicle subtraction osteotomies for fixed sagittal imbalance. Spine. 2003;28(18): 2093–101.10.1097/01.BRS.0000090891.60232.70. 10.1097/01.BRS.0000090891.60232.70 14501920

[7] Smith JS, Sansur CA, Donaldson WF (2011). Short-term morbidity and mortality associated with correction of thoracolumbar fixed sagittal plane deformity: a report from the Scoliosis Research Society Morbidity and Mortality Committee. Spine.

[8] Phan K, Maharaj M, Assem Y (2016). Review of early clinical results and Complications associated with oblique lumbar interbody fusion (OLIF) [J]. J Clin Neurosci.

[9] Ohtori S, Orita S, Yamauchi K (2015). Mini-open Anterior retroperitoneal lumbar Interbody Fusion: oblique lateral Interbody Fusion for lumbar spinal degeneration Disease [J]. Yonsei Med J.

[10] Li JX, Phan K, Mobbs R (2017). Oblique lumbar Interbody Fusion: technical aspects, operative outcomes, and Complications [J]. World Neurosurg.

[11] Hills JM, Yoon ST, Rhee JM (2019). Anterior column Realignment (ACR) with and without Pre-ACR posterior release for fixed Sagittal Deformity. Int J Spine Surg.

[12] Akbarnia BA, Mundis GM, Moazzaz P (2014). Anterior column realignment (ACR) for focal kyphotic spinal deformity using a lateral transpsoas approach and ALL release [J]. J Spinal Disord Tech.

[13] Glassman SD, Hamill CL,Bridwell KH et al. The impact of perioperative Complications on clinical outcome in adult deformity Surgery [J]. Spine (Phila Pa 1976). 2007;32:2764–70.10.1097/BRS.0b013e31815a7644. 10.1097/BRS.0b013e31815a7644 18007258

[14] Wewel JT, Godzik J, Uribe JS (2019). The utilization of minimally invasive Surgery techniques for the treatment of spinal deformity [J]. J Spine Surg.

[15] Pellisé F, Vila-Casademunt A, Ferrer M (2015). Impact on health related quality of life of adult spinal deformity (ASD) compared with other chronic conditions [J]. Eur Spine J.

[16] Ailon T, Shaffrey CI, Lenke LG et al. Progressive Spinal Kyphosis in the Aging Population [J]. Neurosurgery. 2015, null: S164-72. 10.1227/NEU.0000000000000944. 10.1227/NEU.0000000000000944 26378354

[17] Liu H, Yang C, Zheng Z (2015). Comparison of Smith-Petersen osteotomy and pedicle subtraction osteotomy for the correction of thoracolumbar kyphotic deformity in ankylosing spondylitis: a systematic review and meta-analysis. Spine.

[18] Neal CJ, McClendon J, Halpin R (2011). Predicting ideal spinopelvic balance in adult spinal deformity [J]. J Neurosurg Spine.

[19] Logroscino CA, Proietti L, Pola E et al. A minimally invasive posterior lumbar interbody fusion for degenerative lumbar spine instabilities [J]. Eur Spine J, 2011, null: S41-5.10.1007/s00586-011-1762-1. 10.1007/s00586-011-1762-1 PMC3087039 21445617

[20] Barone G, Scaramuzzo L, Zagra A (2017). Adult spinal deformity: effectiveness of interbody lordotic cages to restore disc angle and spino-pelvic parameters through completely mini-invasive trans-psoas and hybrid approach [J]. Eur Spine J.

[21] Saigal R, Mundis GM, Eastlack R et al. Anterior column realignment (ACR) in adult sagittal deformity correction: technique and review of the literature [J]. Spine (Phila Pa 1976), 2016, null: S66-73. 10.1097/BRS.0000000000001483. 10.1097/BRS.0000000000001483 26839994

[22] Cheung ZB, Chen DH, White SJW (2019). Anterior column Realignment in adult spinal deformity: a Case Report and Review of the literature [J]. World Neurosurg.

[23] Mundis GM, Turner JD, Kabirian N (2017). Anterior column Realignment has similar results to Pedicle Subtraction Osteotomy in treating adults with Sagittal Plane deformity. World Neurosurg.

[24] Pimenta L, Oliveira L, Schaffa T (2011). Lumbar total disc replacement from an extreme lateral approach: clinical experience with a minimum of 2 years’ follow-up [J]. J Neurosurg Spine.

[25] Rodgers WB, Gerber EJ, Patterson J (2011). Intraoperative and early postoperative Complications in extreme lateral interbody fusion: an analysis of 600 cases [J]. Spine (Phila Pa 1976).

[26] Cummock MD, Vanni S, Levi AD (2011). An analysis of postoperative thigh symptoms after minimally invasive transpsoas lumbar interbody fusion [J]. J Neurosurg Spine.

[27] Uribe JS, Isaacs RE, Youssef JA et al. Can triggered electromyography monitoring throughout retraction predict postoperative symptomatic neuropraxia after XLIF? Results from a prospective multicenter trial [J]. Eur Spine J, 2015, null: 378 – 85.10.1007/s00586-015-3871-8. 10.1007/s00586-015-3871-8 25874744

[28] Rao PJ, Loganathan A, Yeung V (2015). Outcomes of anterior lumbar interbody fusion Surgery based on indication: a prospective study [J]. Neurosurgery.

[29] Gagliardi MJ, Guiroy AJ, Camino-Willhuber G (2023). Is Indirect Decompression and Fusion more effective than Direct Decompression and Fusion for treating degenerative lumbar spinal stenosis with instability? A systematic review and meta-analysis. Global Spine Journal.

[30] Li J, Li H, Zhang N (2020). Radiographic and clinical outcome of lateral lumbar interbody fusion for extreme lumbar spinal stenosis of Schizas grade D: a retrospective study [J]. BMC Musculoskelet Disord.

[31] Schizas C, Theumann N, Burn A (2010). Qualitative grading of severity of lumbar spinal stenosis based on the morphology of the dural sac on magnetic resonance images [J]. Spine (Phila Pa 1976).

